# Metabolic/bariatric surgery optimization: a position statement by Arabic association for the study of diabetes and metabolism (AASD)

**DOI:** 10.1186/s13098-024-01564-w

**Published:** 2025-01-29

**Authors:** Amin Roshdy Soliman, Hesham Magd eldin Saleem, Amr Abel Hady El Meligi, Mervat Naguib, Rasha Sobh Mohamed, Ghada Rabie Abdelaziz, Maha Rakha, Shereen Abdelghaffar, Abd ElKhalek Hamed, Hany Abd El Rahman saeed Hammad, Eman O Mahmoud, Inass Shaltout

**Affiliations:** 1 Faculty of Medicine,Cairo University, Nephrology Department, Cairo, Egypt; 2 Faculty of Medicine,Cairo University,Internal Medicine Diabetes and Endocrinology Department, Cairo, Egypt; 3 Faculty of Medicine,Cairo University, Pediatric Diabetes and Endocrinology Department, Cairo, Egypt; 4 Military Medical Academy, internal medicine Department, Cairo, Egypt

**Keywords:** Bariatric surgery, Metabolic surgery, Outcomes, Type 2 diabetes mellitus, Weight loss, Minimally invasive bariatric surgery, Laparoscopic bypass, Revisional bariatric surgery, Gastric Band, Gastric bypass, Sleeve Gastrectomy, Weight loss, Operations for obesity, Endoscopic approaches, Anti-obesity medications

## Abstract

For patients considering bariatric surgery, it is essential to have clear answers to common questions to ensure the success of the procedure. Patients should understand that surgery is not a quick fix but a tool that must be complemented by lifestyle changes, including dietary adjustments and regular physical activity. The procedure carries potential risks that should be weighed against the potential benefits. Health authorities play a critical role in ensuring that bariatric surgery is performed under the highest standards of care. Recommendations are provided to determine who is an appropriate candidate for surgery, what preoperative evaluations are necessary, and how to monitor patients postoperatively to maximize outcomes and minimize risks. Additionally, authorities are responsible for ensuring access to follow-up care, including nutritional support and psychological counseling, which are vital for the long-term success of bariatric surgery.

Understanding these aspects by both patients and decision-makers is critical before proceeding with bariatric surgery. The following questions guide patients and healthcare professionals in making informed decisions about the procedure and managing the expectations and outcomes associated with bariatric surgery.

## Introduction

The global prevalence of obesity and its related health complications is on the rise. According to the WHO, approximately 1in 8 people in the world live with obesity. In 2022,2.5 billion adults (18years and older) were overweight. Of these,890 million were living with obesity. In the Middle East and North Africa (MENA) region, obesity rates have surged over the past three decades [1, 2], particularly in Gulf countries, Egypt, Libya, Lebanon, and Iraq, where the prevalence exceeds 30% [3].

Obesity has been linked to the development of multiple diseases, frailty, and reduced quality of life [4]. It significantly raises the risk of developing cardiometabolic conditions, such as coronary artery disease, hypertension, heart failure, dyslipidemia, stroke, type 2 diabetes, musculoskeletal disorders, fatty liver disease, sleep disorders, various cancers, and chronic kidney disease [5]. Research shows that weight-loss interventions, including pharmacotherapy and lifestyle modifications, can significantly reduce the risk of these obesity-related comorbidities [6].

Bariatric surgery, recommended if lifestyle interventions and drug treatment have failed for individuals with a body mass index (BMI) over 40 kg/m² or over 35 kg/m² with obesity-related conditions, has been shown to result in substantial and sustained weight loss and a significant reduction in comorbidities [7]. The procedure has gained prominence due to its effectiveness in improving metabolic profiles [8–10]. However, patients with obesity often present multiple comorbidities that heighten surgical risks, underscoring the need for proper patient selection, thorough preoperative assessments by a multidisciplinary team, and meticulous postoperative care [11].

While the number of bariatric surgeries is increasing in the MENA region, the overall context of these procedures is not well defined [12]. Most surgeries in this region are performed in the private sector, where there is a lack of standardized local guidelines and oversight in terms of multidisciplinary team involvement, patient selection, and proper perioperative care. This may lead to higher rates of surgery-related complications and mortality.

## Rationale and objectives of position Statement

With the rise in obesity and bariatric procedures in Egypt and Arab countries, there has been a surge in the number and types of procedures offered to patients. AASD highlights the importance of ethics in offering these new procedures. Moreover, the AASD reviewed literature to determine when procedures, indications, contraindications, reversals, and corrective surgeries can be offered. There is a need for a position statement from the AASD in response to inquiries made by patients, physicians, society members, hospitals, health insurance payors, the media, and others regarding health optimization of bariatric and metabolic surgery. This statement aims to generate recommendations based on current clinical knowledge, expert opinion, and published peer-reviewed scientific evidence, primarily from our region, to guide health authorities in Egypt and surrounding countries. The statement is not intended to establish a local, regional, or national standard of care and will be revised in the future as additional evidence becomes available.

## Method

The goal of this review is to enhance the effectiveness of bariatric surgery in reducing obesity-related comorbidities among patients in the Arab region and the MENA (Middle East and North Africa) region.

Search Strategy: We conducted a systematic search in PubMed, Cochrane Library, Google Scholar, and Embase using the following terms: ‘bariatric surgery,’ ‘obesity,’ ‘comorbidities,’ ‘Middle East and North Africa,’ ‘Arab countries,’ and ‘weight loss.’ Studies published between 2000 and 2024, in English, involving patients with obesity (BMI ≥ 30 kg/m²) were included. Boolean operators (e.g., AND, OR) were used to combine terms. The criteria for including studies comprised studies and populations from the MENA region and Arab countries. We specifically reviewed randomized controlled trials, medical reviews, and meta-analyses. Two independent reviewers initially screened the titles and abstracts to determine their relevance. Following this, full-text articles of studies deemed potentially relevant were thoroughly assessed. Any discrepancies between the reviewers were resolved through discussions held in group Zoom meetings, ensuring a consensus was reached.

The following questions guide patients and healthcare professionals in making informed decisions about the procedure and managing the expectations and outcomes associated with bariatric surgery.

## Who could benefit from bariatric surgery, and who may not?

Although BMI is not a perfect indicator of adiposity-related chronic disease complications, it is commonly used to classify patients with overweight or obesity. However, BMI can be influenced by factors such as sex, age, ethnicity, and fat distribution. There are no available studies regarding the cutoff value of obesity coming from the MENA region. However, a study published in Lancet Diabetes Endocrinology showed that the cutoff is 30 kg/m2 for the White population: 28.1 kg/m2 for the Black population, 26.9 kg/m2 for the Chinese, 26.6 kg/m2 for the Arab population and 23.9 kg/m2 for South Asian adults [13]. Therefore, defining cutoff values specific to Arabs is essential. Moreover, there are other indicators which might help such as waist circumference, but their significance is not validated [14]. An elegant Chinese study suggested other novel indicators visceral adiposity index (VAI), lipid accumulation product (LAP), and waist circumference-triglyceride index (WTI) for better patient selection [15].

Metabolic surgery is recommended for people with D.M type2 if their BMI ≥ 40 kg/m², and for those with a BMI of 35.0–39.9 kg/m² who could not achieve durable weight loss and improvement in comorbidities. According to the American Diabetes Association (ADA) 2023 recommendations, metabolic surgery may also be considered for adults with a BMI of 30.0–34.9 kg/m² who do not achieve durable weight loss and improvement in comorbidities with nonsurgical methods [16].

The American Association of Clinical Endocrinology (AACE) 2023 recommended metabolic (bariatric) procedures for persons with a BMI of 30–34.9 kg/m²if their DM was uncontrolled despite lifestyle and medical therapy, and for those with a BMI > 35 kg/m² if they have one or more obesity-related complications [17].

The American Society for Metabolic and Bariatric Surgery (ASMBS) and the International Federation for the Surgery of Obesity and Metabolic Disorders (IFSO) 2022 strongly recommend metabolic bariatric surgery (MBS) for individuals with a BMI ≥ 35 kg/m², even in the absence of obesity-related comorbidities. For individuals with class I obesity (BMI 30–34.9 kg/m²) who do not achieve substantial or durable weight loss or comorbidity improvement with nonsurgical methods, MBS should be considered, though medical weight loss is generally more durable for those with a BMI < 35 kg/m² [18].

Obesity-related comorbidities to consider include type 2 diabetes, prediabetes, hypertension, hyperlipidemia, obstructive sleep apnea (OSA), metabolic-associated fatty liver disease, gastroesophageal reflux disease (GERD), asthma, venous stasis disease, severe urinary incontinence, debilitating arthritis, or significantly impaired quality of life [19].

In the elderly, the presence and severity of comorbidities, along with frailty, rather than age alone, are associated with higher postoperative complication rates. Therefore, no specific age cutoff form metabolic bariatric surgery MBS is supported after careful assessment [19].

Obesity is associated with poorer outcomes in some surgeries, such as total joint arthroplasty, abdominal hernia repair, and organ transplantation. Many reports found that these outcomes might improve if they are preceded by MBS in individuals with severe obesity [20].

Although no absolute contraindications to bariatric surgery are confirmed, there are important relative contraindications such as severe heart failure, unstable coronary artery disease, severe renal impairment, active cancer, portal hypertension, alcohol or drugs dependency, cognitive impairment, and any contraindications to general anesthesia [21].

## What are the different types of bariatric/metabolic surgical procedures and what are underlying mechanism(s) for weight loss or morbidity recovery after each one in individuals living with obesity?

Bariatric/metabolic procedures are classified according to the underlying mechanism of weight reduction into: Restrictive surgeries as adjustable gastric banding (AGB) and vertical sleeve gastrectomy (VSG), and restrictive/malabsorptive surgeries as Roux-en-Y gastric bypass (RYGB) and biliopancreatic diversion with duodenal switch (BPD/DS). In addition, the one anastomosis gastric bypass (OAGB), also known as Omega Loop/Mini-gastric Bypass, was introduced in 1997 and has grown popular in Europe and Asia [22, 23]. The single anastomosis duodeno-ileostomy with sleeve gastrectomy (SADI-S), a highly malabsorptive procedure similar to BPD/DS, was first described in 2007 [24].

AGB, introduced as the first laparoscopic bariatric procedure in 1993 [25], is associated with less weight loss compared to other bariatric procedures, with high failure (50%) and reoperation (60%) rates. Therefore, the frequency of this surgery has declined significantly in the last years all over the world [26]. In Sleeve gastrectomy (SG), 80% of the stomach is removed, which results in rapid fullness of the stomach and sense of early satiety after eating. After (SG) serum ghrelin, a hormone that increases hunger is reduced, and gastric inhibitory polypeptide (GIP) and glucagon-like peptide-1 (GLP-1) production are increased [27]. These changes stimulate insulin release, delay gastric emptying, and induce satiety, resulting in decreased blood glucose levels in individuals with type 2 diabetes [27]. Additionally, changes in gut microbiota after VSG impact the gut-brain axis, further reducing appetite. So, VSG cannot be considered only a restrictive procedure but a metabolic procedure with other multiple physiological effects [28].

Restrictive/malabsorptive procedures, such as RYGB, BPD/DS, OAGB and SADI-S reduce stomach capacity and the absorptive surface, which is significantly smaller after BPD/DS. In OAGB procedure, a long and narrow lesser-curvature gastric pouch is created and anastomosed to the jejunum after bypassing 150 to 200 cm of the jejunum [22]. SADI-S was designed to replace the complex surgery, BPD-DS with a more simple and safer procedure [20] especially in individuals with BMI 50 kg/m2 or more and in high-risk surgical patients [29–32]. These procedures affect hormones, gastro-intestinal peptides, and intestinal microbiota, playing a crucial role in weight loss and diabetes remission. After RYGB leptin levels are reduced. Also, GLP-1 levels after food intake are higher in people who have had RYGB than in those who have had VSG I year after surgery. Nowadays VSG followed by RYGB are the most frequently performed bariatric surgeries worldwide [33]. Currently, most bariatric surgeries are performed laparoscopically or robot-assisted due to lower mortality, fewer complications, shorter hospital stays, and faster recovery compared to open surgery [34].

VSG’s early morbidity is intermediate between AGB and RYGB. VSG is technically easier than RYGB, making it a more safe and suitable choice for high-risk people including older individuals and those with many comorbidities [35, 36].

In people with Obesity IV and V, and those with previous abdominal surgeries, RYGB is technically difficult due to the reduced space for intestine rerouting. Additionally, RYGB may affect the absorption and efficacy of psychiatric medications. So, the non-oral antipsychotic formulations may be preferred in these patients [37]. BPD-DS is a more difficult procedure than other metabolic procedures and is associated with higher early complications, morbidity, and mortality. It is used in only 0.5% of patients [38] and is typically reserved for those with insufficient weight loss after SG [39] or those with severe comorbidities like refractory diabetes [40]. OAGB is technically simple, safe, effective and results in long term weight loss, in addition to easy reversibility when necessary [41].

Weight loss after VSG is intermediate between AGB and RYGB, with RYGB generally resulting in more weight loss and better diabetes improvement. However, some studies have found comparable and significant weight loss after both RYGB and SG at 6 and 12 months of follow-up [42, 43]. Basiony et al. (2020) found more rapid weight loss was achieved in a higher percentage of patients after VSG compared to RYGB at 6 months after surgery, while RYGB resulted in a significantly higher percentage of excess weight reduction one year after the procedure. VSG and RYGB had equal effects on the resolution of the different components of metabolic syndrome [44]. Worsening or the appearance of new-onset gastroesophageal reflux disease (GERD) is higher after VSG than RYGB. VSG cannot be reversed due to the extensive removal of stomach. RYGB can be reversed, if necessary, though it is challenging. The results of the Byband sleeve trial should be published soon. These preliminary presentations at conferences challenge the view that sleeve gastrectomy and RYGB give similar outcomes. OAGB achieves 30–40% body weight reduction and diabetes improvement comparable to or superior to RYGB [45]. SADI-S produces weight loss similar to RYGB initially but better long-term, with metabolic effects like biliopancreatic diversions. SADI-S achieves 60–80% T2D remission, with high improvement rates in dyslipidemia (> 70%), OSA (> 80%), and hypertension (> 50%) [29, 30]. It shows good outcomes both as primary surgery and revision after failed procedures, particularly VSG [46, 47].

## How does a multidisciplinary approach improve the effectiveness and safety of preoperative management for bariatric surgery?

Multidisciplinary team (MDT) management is recommended by many medical societies to maximize benefits and prevent or treat complications [48, 49]. However, studies indicate inconsistencies in preoperative preparation for surgery in the MENA region [50]. Significant variations in these conducts were found in a survey performed by the Arab Society of Metabolic and Bariatric surgery (PASMBS) [51]. While opinions on the makeup of MTDs vary widely, many institutions have the following essential members.

1. Metabolic Physician: Specializes in obesity assessment, including related comorbidities and endocrine disorders. This role involves selective referrals for conditions such as obstructive sleep apnea, reflux, or heart disease that may require preoperative evaluation [52]. The metabolic physician also manages diabetes optimization pre- and post-surgery according to guidelines [53].
2. Bariatric Surgeon: Determine the type of bariatric surgery in conjunction with the MDT, evaluates surgical suitability, and advises patients of risks, advantages and options [55]. More than 60% of PASMBS surgeons perform over 125 cases annually, though most (63.4%) are in private practice. If these surgeons have a multidisciplinary team, it’s unclear [54].
3. Nutritionist: Offers a diet low in calories and Liver-reduction prior to surgery [55], maintains weight over the long term, and monitors micronutrient deficits after surgery [56]. There aren’t many published guidelines for dietary care to far obesity: identification, assessment and management [57], although one Saudi Arabian study stands out [58].
4. Psychiatrist: Crucial for treating mental illnesses and unhealthy eating habits [59]. Preoperative psychiatric examination is advised by both the American Society for Metabolic and Bariatric Surgery [61] and the British Obesity Metabolic Surgery Society [60] with referrals to a psychiatrist only for substantial abnormalities. Post-operative psychiatric support is also important. A study in the Middle East showed only 22.6% of patients were referred to a psychologist [51].
5. Anesthetist: Plays a crucial role in assessing patients with significant medical comorbidities before surgery and during the perioperative period [52].
6. Gastroenterologist: Useful for preoperative endoscopy and managing postoperative conditions like severe reflux disease or anastomotic aperture reduction [62]. The Saudi Arabian Society for Metabolic and Bariatric Surgery advises routine preoperative endoscopy as part of its guidelines [63].
7. Data Registry: Tracks baseline obesity-related conditions, operation types, operative outcomes, and disease status post-surgery. Kuwait was the first to establish a national report on metabolic surgery in the Arabian region.

## How could the potential risks of complications from bariatric surgery balance against its long-term benefits for health and weight control?

The spectrum of complications that can occur after bariatric surgery is wide and varied [64]. Major morbidity in bariatric surgery occurs in approximately 13% of cases, with complication rates of 8–13% for laparoscopic sleeve gastrectomy [65] and 3-12.5% for laparoscopic gastric bypass (RYGB) [66]. Intraoperative complications include anastomotic leaks, the most serious issue, which can increase overall morbidity to 61% and mortality to 15% [67]. Stenosis after RYGB occurs in 8–19% of cases [68], and symptoms such as dysphagia may result from kinks or volvulus around the sleeve gastrectomy (SG). These complications could occur in up to 9% of patients [69]. Postoperative bleeding which requires intervention occurs in up to 11% of cases for both RYGB and SG. There is “early” dumping syndrome and “late” dumping, also known as reactive hypoglycemia or post-prandial hypoglycemia. The symptoms can present in the sleeve gastrectomy and RYGB [70, 71] Other potential challenges include infection at surgical sites and adverse reactions to anesthesia. The early postoperative period is marked by significant physiological adjustments and potential complications, including nausea, vomiting, constipation, and, in 8.6% of cases, new onset gastroesophageal reflux disease (GERD) after sleeve gastrectomy. A recent meta-analysis found that 11.6% of patients develop Barrett’s esophagus, a premalignant condition requiring endoscopic surveillance [72]. Patients may also experience pain, often managed with opioids, but new research suggests patients following bariatric surgery may be more prone to chronic opioid use, particularly those with postoperative complications or less weight loss [73]. Other problems may include anastomotic leaks, strictures, infections, bleeding, renal stones, and osteoporosis. Although venous thromboembolism rates are low after bariatric surgery, pulmonary embolism remains a common cause of mortality [74].

Individuals who are eligible for bariatric surgery frequently struggle with mental health conditions such as depression and anxiety. They may also experience specific eating disorders, particularly binge eating. The prevalence of these psychological issues in this population exceeds 15%. A recent study found that the relative risk of suicide was 64% higher in the surgical groups compared to the nonsurgical groups [75]. Late postoperative complications that occur months or years after surgery include Cholelithiasis and gallstone formation following gastric bypass surgery, ranging from 32 to 42% [76]. Patients could present with stones in the common bile duct, and this could result in cholangitis or pancreatitis. The incidence of small bowel obstruction (SBO) is between 1.5% and 5% [77], which is attributed to internal hernias, adhesions, or strictures.

Protein-caloric malnutrition is a major complication of BPD-DS but less common in other restrictive/malabsorptive procedures [29, 30]. All bariatric procedures can lead to deficiencies in vitamin B12, folate, iron, vitamin D, and minerals like zinc, selenium, and copper. OAGB, BPD/DS, and SADI-S also increased risk of fat-soluble vitamin deficiencies. While vitamin supplementation is necessary after all procedures, OAGB with long biliopancreatic limb, BPD/DS, and SADI-S require higher doses of fat-soluble vitamins, minerals, and protein. Thiamine deficiency can also occur after metabolic and bariatric procedures due to poor nutrition, rapid weight loss, supplement non-adherence, or vomiting [78, 79].

Vitamin B12 deficiency affects 30–35% of patients one year postoperatively. This can lead to megaloblastic anemia and neurological symptoms such as paresthesia (tingling and numbness), impaired balance, memory problems, agitation, confusion, and depression. Low serum folate after RYGB occurs in 6–35% of patients and can cause megaloblastic anemia, irritability, poor memory, or paranoid behavior. Thiamine deficiency is a serious concern, potentially causing dry beriberi, wet beriberi, or Wernicke’s encephalopathy. Iron deficiency is the most common nutritional deficiency post-RYGB, affecting 30–50% of patients and causing fatigue and generalized weakness. There is also a high prevalence of vitamin D deficiency, which can lead to secondary hyperparathyroidism, calcium malabsorption, osteopenia, and osteoporosis [80].

A 2020 survey by the Pan-Arab Society for Metabolic and Bariatric Surgery showed that 70 patients had morbidity (33 with leaks, 23 with venous thromboembolism (VTE), and 15 requiring reoperation) or mortality. The overall rates of leaks, VTE, reoperations, and mortality were 1.96%, 1.2%, 1.5%, and 0.5%, respectively. Mortality was higher after complications (46.9% overall, 53.8% after reoperation, 42.9% after VTE, and 24.1% after leaks). Sleeve gastrectomy was the most common procedure associated with complications (58.6%) but had the lowest mortality rate after any complication (35% vs. 57–60% for other procedures) [81]. Studies indicate that there is an increased incidence of complications in the MENA region, most probably due to a lack of knowledge about bariatric surgery among both doctors and patients.

## In what situations might bariatric surgery reversal be necessary, and what are the potential outcomes?

Some of patients living with obesity patients undergoing bariatric/metabolic surgery, may experience recurrent or persistent disease or suffer severe complications. These patients might require an escalation of therapy or a new treatment modality [82]. Bariatric reoperations address either Suboptimal Clinical Response (SoCR), Recurrent Weight Gain (RWG), or procedure complications like reflux, leaks, and malnutrition. These secondary surgeries include: revisional procedures that modify without changing the mechanism, conversion procedures that switch to a different mechanism, and reversal procedures that restore normal anatomy [83].

Studies from the MENA region indicate that weight regain after bariatric surgery ranges from 5 to 39%, varying according to the type of surgery and patient compliance. The incidence of revisional procedures after primary bariatric surgery has been estimated between 3% and 60%, depending on the primary operation. As an example, the rate of revisional surgery is 10.5-60% after laparoscopic adjustable gastric banding (LAGB) and about 38.5% at 10 years after sleeve gastrectomy (SG) [84–87]. In 2016 the International Federation for the Surgery of Obesity and Metabolic Disorders (IFSO) declared that revisional surgery resembled 7.4% of all bariatric procedures globally [88]. Most revisional bariatric surgeries (91.3%) involved conversion from sleeve gastrectomy to gastric bypass [88]. When compared to primary bariatric surgery, revisional surgery carries a higher risk of perioperative complications. Thus, multidisciplinary assessment, including radiological and endoscopic studies, together with the evaluation of nutritional and behavioral status, is recommended before doing revisional surgery [89].

From the different bariatric procedures, Roux-en-Y gastric bypass (RYGB) has the higher results regarding loss of excess weight and resolution of comorbid conditions. It is often the procedure of choice for revising failures from other primary bariatric [90]. However, RYGB can also lead to life-threatening complications, such as dumping syndrome, malnutrition, refractory hypoglycemia, and hypocalcemia, which may result in a necessary reversal procedure [91, 92]. Up to 25% of patients undergoing bariatric surgery may need a second operation for complications [93]. The prevalence of reversal of one anastomosis gastric bypass (OAGB) to normal anatomy is 1%, with the main indicators including protein-energy under nutrition and hypoalbuminemia. The hospitalization rate due to protein malnutrition is 1% annually, associated with poor patient health outcomes. RYGB reversal has three times higher incidence of complications than OAGB reversal (29% vs. 10.9%), possibly due to the less technically challenging nature of OAGB procedures [94, 95].

Regarding GLP-1 receptor agonist (GLP-1-RA) versus revisional surgery: GLP-1RA show promise as a potential solution for post-bariatric surgery WR and IWL. The use of GLP-1RA can safely lead to loss of two thirds of the weight regain. liraglutide and semaglutide has shown promising outcomes, as use of liraglutide led to 7.3% total weight loss (TWL) and use of semaglutide resulted in 9.8% TWL [96]. In a retrospective cohort study of patients who underwent SG and sought treatment for weight recurrence, treatment with semaglutide and tirzepatide resulted in clinically significant mean weight losses of 10.3% and 15.5%, respectively, with no reported severe adverse events [97].

## What is the effect of bariatric surgery on type 2 diabetes remission and long-term blood sugar control?

Many studies have linked obesity, type 2 diabetes T2DM, and body weight. These studies have demonstrated that early weight-loss studies highlighted that early weight-loss interventions in prediabetic patients significantly decrease the development of type 2 diabetes [98]. Bariatric surgery has been investigated as a method for diabetes prevention. A 2021 observational longitudinal study evaluated its effectiveness in 699 patients who had both severe obesity and prediabetes and underwent this surgical intervention. In this study, prediabetes was characterized by a baseline glycated hemoglobin (A1c) level between 5.7% and 6.4%, with participants not on any anti-diabetes medications. The study population had an average age of 45.4 years, a body mass index (BMI) of 43.8 kg/m², and a median A1c of 5.9%. The postoperative prediabetes remission rates were 82%, 73%, 66%, and 58%, respectively, at the 1st, 2nd, 3rd, and 4th years of follow-up. Younger patients presented a higher remission rate. The remission rate decreased with the follow-up period [99].

In 2024 the ADA updated the definition of diabetes remission to a HbA1c less than 6.5% for at least 3 months after suspending oral hypoglycemic drugs [100]. Diabetes duration is a key factor in the diabetes remission rate and persistence. Diabetes remission scores have been used to predict remission following metabolic surgery. Examples of which are the DiaRem score (includes age, diabetes treatment, and HbA1c) and the ABCD score (age at operation (A), baseline BMI (B), C-peptide level (C), and diabetes duration (D) [101]. Additional baseline characteristics that positively predict diabetes remission include better glucose control and better β-cell function. Weight loss after surgery has been demonstrated to be one of the most important factors that increase the chance of achieving better diabetes control [102]. This partially explains the seemingly higher success rates of diabetes remission post-metabolic surgery compared to conventional methods, as a higher percentage of studies were performed on patients with diabetes with a shorter disease duration and better preoperative diabetes control. Randomized control trials have focused on the question of sustainability of diabetes remission after metabolic surgery in comparison to conventional measures for weight loss. For example, in 2021 Mingrone et al. performed an open-label, randomized control study including 60 T2DM patients with a BMI of 35 kg/m² and above, comparing the effects of RYGB, BPD, and medical management on 10-year diabetes remission. Although the results supported an initial higher rate of diabetes remission in the surgical group (50.0% for BPD and 25.0% for RYGB), 58.8% of patients experienced a relapse during the follow-up period [103].

An Egyptian prospective multi-centric study conducted in 2020 included 250 patients with obesity who underwent bariatric surgery and completed 1 year of postoperative follow-up. Of these, 209 patients had diabetes prior to the intervention. The patients were evaluated prior to surgery and again at 1, 6, and 12 months postoperatively, and results showed that remission occurred in nearly 90% of patients. The mean age of the patients was 34 years, suggesting a likely short duration of diabetes, though this was not clarified in the study [104]. Another retrospective study including diabetic patients in Saudi Arabia and Egypt who underwent gastric sleeve surgery showed better diabetes control in Saudi patients compared to Egyptians, there was an improvement in blood sugar readings of 57.1% versus 23.8%. The mean age of Egyptian patients and the duration of diabetes among them were higher than those of Saudi patients [105].

Semaglutide and tirzepatide, a dual analogue of GLP-1and glucose-dependent insulinotropic polypeptide (GIP) receptor agonist, have been added to the pharmaceutical arsenal against T2DM. Two phase 3 trials have addressed the potential positive effect of the dual GIP and GLP-1 receptor agonist (tirzepatide) for obesity (SURMOUNT-1, patients with obesity only, and SURMOUNT-2, for patients with obesity and type 2 diabetes). In the SURMOUNT-2 trial, tirzepatide resulted in body weight loss of 9.6% and 11.6% more than placebo, and an A1C lowering of 1.55% and 1.57% more than placebo after 72 weeks of treatment with the 10 mg and 15 mg doses. Tirzepatide (TZP) resulted in impressive remission rates ranging from 66 to 81% after 52 weeks, depending on the drug dosage [106, 107]. The SURPASS program of randomized clinical trials provided evidence of TZP’s efficacy and safety in various clinical settings. TZP improved patients’ quality of life by achieving multiple targets in T2D, such as lower HbA1c levels and greater body weight loss [108].

## What are the potential benefits and risks of bariatric surgery for children and adolescents?

Globally, it is estimated that 400 million children and adolescents suffer from obesity [109, 110]. On a national level, 22.7% of children aged 10–19 years and 37.5% of those aged 5–19 years are patients with obesity, according to the Global Health Survey in Egypt 2021 [111].

It is widely accepted that the most severe forms of pediatric obesity (i.e., class II obesity; BMI ≥ 35 kg/m², or 120% of the 95th percentile for age and sex, whichever is lower) lead to many related comorbidities, with both increased morbidity and mortality [112]. Hence, bariatric surgery has been considered for adolescents with severe obesity, 13 years or older after other lines of therapy have failed [113]. Observational studies have compared adolescent cohorts undergoing bariatric surgical treatment versus intensive obesity treatment or nonsurgical controls [114]. These studies suggest that weight loss surgery is safe and effective for pediatric patients with class II or class III obesity, but only if performed in comprehensive metabolic and bariatric surgery settings with experience in working with youth and their families [114]. The two most performed procedures in adolescents are LSG (laparoscopic sleeve gastrectomy) and RYGB (Roux-en-Y gastric bypass). In the Teen-LABS cohort, RYGB and LSG groups experienced a mean weight reduction of 27% and resolution of comorbidities at 3 years, including type 2 diabetes mellitus (95%), hypertension (74%), and dyslipidemia (66%), with an accompanying reduction in the prevalence of multiple concurrent cardiovascular disease risk factors (3 or more) [115].

In Egypt, at Ain Shams University, a study by Zakaria and Matar in 2018 found that LSG when performed in adolescents, resulted in excess body weight loss (%EWL) of 50.2% ± 19.3% and 73.3% ± 20.1% at six months and one year, respectively. The mean postoperative BMI was 33.8 ± 6.8 kg/m² and 27.6 ± 5.1 kg/m² at six months and one year, respectively [116].

The link between obesity and type 2 diabetes in children and adolescents has been well established, moreover, overweight and obesity are also common among people with type 1 diabetes (T1D) [117]. In a study conducted by Liu et al., it was reported that the prevalence of obesity and overweight among U.S. young patients with type 1 diabetes aged 3 to 19 years was 12.6% and 22.1%, respectively [117]. A systematic review in June 2021 by Grabia et al. found that overweight and obesity in children and adolescents with T1DM averaged 20.1% and 9.5%, respectively, moreover, there was increasing incidence of metabolic syndrome, ranging from 3.2 to 29.9%, depending on the criteria used [118].

Information on bariatric surgery (BS) in children and adolescents are scarce. Moreover, data concerning patients who passed into adulthood with type 1 diabetes (T1D) are limited; largely consisting of case series with a small number of patients and several case reports that present inconsistent findings. Some studies have indicated a reduction in body mass index (BMI) of 11 to 13 kg/m² and a decrease in total daily insulin requirements in adult patients with obesity and T1D, with an average change of 0.34 units/kg/day. Moreover, studies with small sample sizes have found improvements in comorbidities, such as decreases in systolic and diastolic blood pressure as well as indices of lipid profiles in adult people living with type1 diabetes [117].

Some research suggests that even adults with long-standing type 1 diabetes (T1D) may still have some ongoing regeneration of islet cells. The timing of bariatric surgery may be important in preserving beta cell mass and preventing the progression to complete insulin deficiency. GLP-1 receptor agonists (GLP-1RA) may help protect beta cell mass and slow the progression of the disease if surgery is performed early in its course [119, 120]. A systematic review with meta-analysis reported that in adult people with T1D, liraglutide might be an adjunct to insulin, improving glycemic control, inducing body weight loss, and decreasing exogenous insulin requirements and severe hypoglycemia in people with T1D.In these patients, careful monitoring of blood glucose level or use of continuous glucose monitoring can increase safety of use GLP1RA for treatment of obesity [121].

On the other hand, adverse events have been documented in T1D adult patients with obesity who underwent BS. There is a considerable risk of diabetic ketoacidosis (DKA) and severe hypoglycemic episodes requiring hospitalization. Aminian et al. reported that the incidence of DKA in the early postoperative period (0–61 days) in people with T1D is as high as 25%. The main precipitating factors leading to DKA were found to be altered glucose kinetics, poor perioperative glycemic control with discontinuation or inadequate intake of insulin, and other factors such as fluid and calorie deprivation, anesthesia, surgical stress and infection [122].

Another relatively frequent complication in T1D patients with obesity undergoing BS is hypoglycemia, with an incidence of up to 70%. Rapid delivery of carbohydrates to the jejunum with disturbed glucose dynamics creates an imbalance between higher and earlier glucose [123]. In the population with type 1 diabetes (T1D), a prevalent issue is the occurrence of autonomic neuropathy and gastroparesis, which can result in complications like nausea, vomiting, and acute dilation of gastric remnants [124].

Nutritional deficiencies are a significant concern following metabolic bariatric surgery in adolescents and may lead to osteoporosis, chronic anemia, and/or permanent neurological deficits if unrecognized or inadequately managed [125]. Deficiencies in iron, vitamins B12, A, D, B1, folate, and albumin levels have been reported in adolescents three years after Roux-en-Y gastric bypass or vertical sleeve gastrectomy [126]. Moreover, deficiencies may progress over time as nutrient stores are further depleted; therefore, lifelong micronutrient supplementation is recommended, along with careful monitoring and follow-up with a nutritionist. This is particularly complicated in adolescence, which is a critical stage for biological development and body anabolism. Additionally, lower adherence to nutritional supplementation among youth and the anticipated longer lifespan with altered gastrointestinal physiology may increase the risk of adverse nutritional outcomes [127].

GLP1RA, mainly Liraglutide, approved by food and drug administration FDA in adolescents with obesity from the age of 12 in those over 60 kg or having BMI equivalent to 30 mg/m2, can be an alternative add on therapy, in those with detectable c- peptide levels, and/or infrequent hypoglycemia, with failure of other lines of therapy for at least 6 months. In these patients, careful monitoring of blood glucose level or use of continuous glucose monitoring can increase safety of use of GLP1RA for treatment of obesity [121].

## Can bariatric surgery effectively reverse non-alcoholic fatty liver disease (NAFLD) in patients with obesity?

Patients with stage2-3 non-alcoholic steatohepatitis (NASH) and a BMI of 35 to 40 kg/m² are considered to benefit from bariatric surgery [128], and it is also considered a first-line option if the BMI exceeds 50 kg/m² [129]. Bariatric surgery is shown to be more effective than lifestyle interventions and optimized medical therapy in the treatment of NASH [130].

Bariatric surgery can resolve NASH, improve hepatic fibrosis (with grade 2 or 3 fibrosis being resolved or improved in 60% of patients [131], induce sustained weight loss of up to 30%, cure diabetes, and decrease all-cause morbidity and mortality, with benefits observed five years after [132]. However, some studies have reported worsening of fibrosis [133].

Studies from Egypt [134, 135] have shown consistent results with international findings, with meta-analyses [136, 137] suggesting that resolution of hepatic steatosis, steatohepatitis, and fibrosis was observed in over 75% of patients, with different surgical procedures and a reduction in BMI of 13.4 ± 7.4 kg/m² (*P* = .017) [138].

According to Egyptian clinical practice guidelines [139], bariatric surgery can be offered to patients with metabolic fatty liver disease (MAFLD) only if the following two criteria are met BMI > 40 kg/m² or BMI > 35 kg/m² with obesity-related comorbidities and absence of decompensated cirrhosis or evidence of concomitant portal hypertension (B1). The feasibility of bariatric surgery for patients with BMI ≤ 35 kg/m² are currently unclear.

MBS can reduce MAFLD progression to cirrhosis and potentially improve liver histology and reverse fibrosis. However, for patients with decompensated cirrhosis, MBS carries a high perioperative mortality risk. Careful Patient selection and precise procedure choice are critical for achieving optimal outcomes [140].

In hepatic cirrhosis and liver Transplant, there is evidence that bariatric surgery can be performed safely in selected patients with well-compensated cirrhosis and no evidence of severe portal hypertension. There have been perioperative deaths (3.2%) [141]. In a recent Egyptian study of 132 cases with Child-A MAFLD-related cirrhosis, laparoscopic sleeve gastrectomy (LSG) was found to be safe and led to improvements in steatosis, steatohepatitis, and fibrosis [141]. Bariatric interventions in liver transplant recipients would positively impact their metabolism and liver pathophysiology [142].

In 2022AACE recommend the use of GLP-1RA in obesity proven NASH, elevated probability of NASH or in NAFLD. GLP-1RA reduces hepatic steatosis, BMI, and liver enzymes. However, GLP-1RA may be associated with adverse events compared to other interventions [143]. Tirzepatide, a GIP/GLP-1RA, in phase 3 clinical trials at 10 mg and 15 mg doses resulted in reductions in liver fat content of 8.21% and 7.78%, respectively, over a 52-week period. Additionally, Tirzepatide significantly decreased the activity of ALT and AST from baseline at 26 weeks in a phase 2 study [143]. In a more recent study, the use of tirzepatide, administered once weekly subcutaneously for 52 weeks, in patients with or without type 2 diabetes mellitus was associated with statistically significant improvements in metabolic dysfunction-associated steatohepatitis (MASH), without worsening fibrosis, in a phase 2 trial of patients with MASH and moderate to severe fibrosis (BMI between 27 and 50 kg/m²). The secondary endpoint, assessed at week 52, was an improvement of ≥ 1 fibrosis stage without worsening of NASH. The mean percentage change in body weight was − 10.7%, − 13.3%, and − 15.6% in the 5 mg, 10 mg, and 15 mg tirzepatide groups, respectively, compared with − 0.8% in the placebo group [144]. The ADA 2024 guidelines prescribe GLP-1RA (semaglutide and tirzepatide) for managing NASH in diabetes.

We can conclude that treatment for obesity is evolving with the introduction of new antidiabetic (weight-loss) drugs. Bariatric surgery remains the most effective treatment for severe obesity, but treatment approaches mostly will include a combination of drugs for obesity, either before or after surgery, especially if patients have not lost enough weight or have regained weight.

## Can bariatric surgery improve both cardiovascular and renal health, and how?

There is clear evidence of a strong relationship between obesity and unfavorable renal outcomes. Obesity is associated with a high risk of chronic kidney disease (CKD), end-stage renal disease (ESRD), as well as renal stone formation and renal cell carcinoma [145]. Suggested mechanisms accounting for this increased risk include obesity-mediated diabetes, glomerular hyperfiltration, renal angiotensin-aldosterone system (RAAS) activation, hypertension, adipocytokine decontrol, inflammation and insulin resistance [146]. There are numerous trials documenting the favorable renal effects of bariatric surgery. In a large retrospective observational study on T2DM patients with obesity, bariatric surgery significantly decreased the incidence of kidney disease that related to nonsurgical interventions (6.4% vs. 14%) [147]. In the MENA region, bariatric surgery was associated with improved kidney disease progression parameters, including a significant reduction in glomerular hyperfiltration and urine protein excretion [148]. Bariatric surgeries may be feasible and safe procedures for selected kidney transplant recipients with obesity. However, there was no significant advantage regarding new-onset diabetes after transplant, total diabetes prevalence, or graft outcomes in the bariatric surgery group compared to the non-surgery group [149]. It is important to note that the renal benefits observed in the aforementioned bariatric surgery studies are related to weight reduction rather than the surgery itself. This is confirmed by a randomized trial in which patients with obesity and diabetic kidney disease were studied after randomization to best medical treatment versus RY gastric bypass. After 5 years of follow-up, remission of albuminuria was not statistically different between the two groups, and about 60–70% of patients achieving remission of microalbuminuria in the two groups [150]. Liraglutide in the SCALE Diabetes trial significantly reduced urine albumin excretion compared to placebo [151]. The LIRA-RENAL and LEADER RCTs also highlighted that Liraglutide slowed the onset and progression of CKD [152, 153]. The effect of semaglutide on kidney outcomes was shown by the early terminated FLOW trial. Patients treated with semaglutide experienced a 24% reduction in the risk of the composite primary endpoint, which encompassed kidney outcomes and mortality related to renal and/or cardiovascular (CV) causes, in comparison to those receiving a placebo [154].

Renal complications related to bariatric surgery, include acute kidney injury, renal stone formation, and oxalate nephropathy, especially in types of surgery involving higher degrees of malabsorption [155]. The incidence of postoperative acute kidney injury (AKI) following bariatric surgery reaches up to 6%, which is significantly higher than observed in other general surgeries [156].

Obesity is strongly linked to cardiovascular (CV) morbidity and mortality and is associated with many CV risk factors, including hypertension, diabetes, dyslipidemia, and inflammation [157]. Weight loss is considered a standard recommendation for all patients with cardiovascular disease (CVD) and associated obesity [158]. In a large retrospective analysis, bariatric surgery reduced mortality by nearly 50% and reduced hospital stay for heart failure patients [159]. Bariatric surgery can lead to improvement in CV risk profile, cardiac geometry, and function [160]. This cardiac benefit was related to weight reduction rather than bariatric surgery itself, as shown in many trials. In the SELECT trial non-diabetic patients with CVD who received once weekly subcutaneous injection of semaglutide achieved superior results in reduction of the incidence of CV death, nonfatal myocardial infarction, or nonfatal stroke compared to placebo at a follow-up period of about 3years [161]. In the FLOW trial, patients who received semaglutide had a 24% risk reduction of death due to renal and/or CV mortality compared to placebo. The risk of major CV events was reduced by 18%, with a 20% reduction of the risk of all-cause mortality [154].

Accordingly, the FDA has recently approved semaglutide injection for risk reduction of CV death, MI, and stroke in adults with obesity or overweight associated with CV disease [162]. In a phase 3 clinical trial studying the safety and efficacy of tirzepatide in patients with heart failure with preserved ejection fraction (HFpEF) and obesity, in people with and without T2DM, tirzepatide decreased the risk of heart failure outcomes, including Heart failure hospitalization or ER visits, increasing the dose of diuretic, or CV death, by 38% compared to placebo [163].

Bariatric surgery may be associated with cardiovascular complications, including pulmonary embolism, which can be a major cause of death in this group. The incidence of symptomatic post operative venous thromboembolism VTE following bariatric surgery ranges between 0.3 and 3% [164, 165].

## How could bariatric surgery affect mental health and emotional well-being before and after the surgery?

Bariatric surgery is associated with substantial weight loss and improvement in related health conditions, including Type 2 diabetes. Nonetheless, the pathway to surgery and the subsequent post-operative phase necessitates significant alterations in lifestyle, particularly in relation to dietary adherence, increased physical activity, and, in some cases, medication management [166]. Candidates for bariatric surgery frequently present with intricate mental health challenges, such as depression, eating disorders, and substance misuse, which must be adequately addressed prior to the surgical intervention [167]. Preoperative psychological assessments are essential for identifying unrealistic expectations, psychological contraindications including untreated mental health issues, and the need for supplementary support. While bariatric surgery may lead to enhancements in mental health and psychosocial functioning for certain patients, it also poses potential risks, such as maladaptive eating patterns, concerns regarding body image, substance abuse, and even suicidal ideation [168]. Post-operative hurdles, including weight regain, metabolic alterations, and unrealistic expectations, can further complicate these issues [169, 170].Therefore, the participation of clinical psychologists throughout the pre-operative, and post-operative stages is imperative to effectively manage these psychological risks and provide support to patients throughout their weight-loss journey [171].Weight loss patients should undergo psychosocial evaluation by a licensed professional in a behavioral health field such as psychology, and psychiatry [62].

A study conducted In Saudi Arabia involving 366 participants reported a 23.5% prevalence rate of depression among individuals who have undergone bariatric surgery. The severity of depression varied according to the type and timing of the surgical procedure. Among the 313 patients who underwent sleeve gastrectomy, 30.03% indicated experiencing minimal depression, 47.28% reported mild depression, 16.61% moderate depression, 3.51% fairly severe depression, and 2.56% severe depression. Conversely, among the 53 individuals who had gastric bypass surgery, 43.4% reported minimal depression, 28.3% mild depression, 16.98% moderate depression, 11.32% moderately severe depression, and none indicated severe depression [172].

Research by Alabi et al. suggests that nearly half of the candidates for bariatric surgery experience depressive symptoms, which tend to improve post-operatively and stabilize during the first year [173]. However, Martens et al. noted a resurgence of depression rates after the first year, with rates escalating to 8.7% by the second year; further long-term studies are warranted to explore this trend [174].

Additionally, a study conducted in Egypt revealed that 40% of patients exhibited depressive symptoms following surgery, half experienced low self-esteem, and 73% struggled with eating regulation [175]. In the United Arab Emirates, issues related to depression, anxiety, functional impairment, diminished quality of life, and disturbances in self-image were prevalent among 105 patients [176]. In Kuwait, individuals with Type 2 diabetes tended to eschew psychological support due to inadequate services and cultural stigma [177]. In Saudi Arabia, satisfaction levels regarding overall appearance and weight loss after surgery were recorded at 32.3% and 42.6%, respectively, with 78% of respondents expressing interest in pursuing body contouring surgery subsequent to bariatric surgery [178].

GLP-1RA have shown promising results in treating obesity and have also demonstrated potential benefits for several mental health issues [179].

Depression: Studies have reported improvements in depression symptoms in both people with diabetes and non-diabetic patients treated with GLP-1RA.

Anxiety and Stress: Early research suggests a potential reduction in anxiety and stress levels with GLP-1RA treatment, though further investigation is needed to confirm this effect.

Binge Eating Disorder: GLP-1RA have shown success in helping individuals recover from binge eating disorder by curbing cravings and enhancing feelings of fullness.

Alzheimer’s Disease: Preliminary studies suggest GLP-1RA may improve cognitive function, indicating potential benefits for Alzheimer’s patients.

Addictive Behaviors: Research indicates that GLP-1RA not only reduce food cravings but may also help reduce binge drinking, smoking, and other addictive behaviors. These medications appear to affect brain regions involved in mood regulation, neurogenesis, and neuronal function [180].

A meta-analysis of five randomized controlled trials and one prospective cohort study, involving 2,071 participants, showed a significant decrease in depression scale scores for patients treated with GLP-1RAscompared to control groups. However, the most commonly reported side effects of GLP-1RA treatment appetite loss, nausea, vomiting, and diarrhea that may be linked to negative emotions [180].

In conclusion, while bariatric surgery offers significant benefits in weight management and related health problems, MBS comes with psychological and emotional risks that need to be carefully managed that require lifelong lifestyle modification and may present with potential mental health risks, especially for patients with pre-existing psychological disorders. While some experience short-term improvements in mental health post-surgery, there is a risk of depression and maladaptive behaviors emerging or worsening over time, especially after the first year.

## Will obesity medications alter the approach to weight loss surgery?

The field of bariatric surgery is evolving with new anti-obesity medications. The optimal timing and patient selection are needed for combined approach, they can be used before surgery to achieve target weight loss to reduce surgical risks, to help identify patients who are good responders to medical therapy versus those who might benefit more from surgery and to serve as a “bridge therapy” while patients await surgery. Moreover, they can be used post-surgery for those patients who had weight regain after bariatric surgery, or when weight regain occurs despite proper diet/exercise adherence and may help patients who don’t achieve adequate weight loss from surgery alone and for benefits beyond weight reduction.

GLP-1RA, used to treat Type 2 diabetes, have been shown to be effective in promoting weight loss in preclinical and clinical studies. In the SCALE trial overweight and candidates with obesity and Type 2 diabetes, subcutaneous daily 3 mg liraglutide resulted in 6% weight reduction after 56 weeks compared with placebo [181]. In nine randomized controlled trials, largest proportion of studies participants achieved 5–10% weight loss [182]. In the STEP 1 trial, non-diabetic adults with a BMI of 30 or more and received once-weekly 2.4 mg semaglutide achieved weight loss of − 14.9%, compared with − 2.4% with placebo group. Semaglutide together with lifestyle intervention was associated with a sustained, clinically relevant reduction in body weight [183]. The SURMOUNT-4 trial evaluated efficacy and safety of tirzepatide in comparison to placebo; by the end of 36 weeks of treatment, participants achieved a mean weight loss of 21.1% [184]. Tirzepatide appears to be the highly effective available weight-reducing drug, producing an average weight loss of 26%. Tirzepatide, a combined GLP-1 and glucose-dependent insulinotropic polypeptide (GIP) receptor agonist, is the most effective currently available GLP-1 receptor agonist for both glycemic control and weight loss in the short and intermediate term. Future agents in this class might leverage additional mechanisms of action for greater efficacy [185]. Other available FDA-approved anti-obesity medications include orlistat, phentermine/topiramate, and naltrexone/bupropion. Orlistat leads to weight loss of 9.6% compared to 5.6% in placebo group, it can lead to possible side effects on the liver. Phentermine/topiramate results in weight loss of 7.8–9.8%, with potential side effects including tachycardia and hypertension, and the safety concerns include suicidal thoughts, glaucoma, and metabolic acidosis. It is contraindicated in patients with coronary artery disease and hyperthyroidism and is not recommended for long-term use more than 12 weeks. Naltrexone/bupropion leads to an average of 5% weight loss compared to 1.8% in the placebo group, and side effects include agitation, mood changes, tachycardia, and hypertension. It may lead to withdrawal symptoms and seizures. It is contraindicated in hypertension, hyperthyroidism, epilepsy, and a history of suicidal thoughts. If less than 5% weight loss is achieved after 12 weeks of use, it should be discontinued as no further benefit is expected. It is not recommended for individuals under 18 years of age, and patients over 65 years may be more sensitive to central nervous system adverse effects [186].

Newer anti-obesity medications are expecting final approval, some of them can lead to up to 24.2% weight loss from baseline. These medications offer a broader potential to be used successfully competing with invasive metabolic surgeries. While some anti-obesity medications can result in a weight reduction of 14.9–26.1% of baseline weight [187], these are compared with the mean weight loss for different metabolic surgeries: RYGB results in a 27.5% weight loss after 4 years and 21% after 10 years, AGB leads to a mean weight loss of 10.6%, and SG can result in a mean weight loss of 17.8% from baseline [188].

On the other hand, the effects of anti-obesity medications versus bariatric surgery on quality of life are examined. Multiple studies demonstrate the beneficial effect of GLP-1RA analog therapy on quality of life, measured with various scales. In a study done on PCOS young women with obesity treated using liraglutide, improvement in their quality of life (QoL) was significant. The SUSTAIN 6 trials with 3,297 participants showed that semaglutide treatment improved health-related quality of life (HRQoL). Analysis of data from 4,725 participants across three randomized trials evaluating liraglutide efficacy and safety proved improvements in metabolic parameters and HRQoL. It also indicated that liraglutide effectiveness is due to weight loss and independent mechanisms beyond weight loss [189].A meta-analysis of 18 published studies on the long-term consequences of bariatric surgery found that HRQoL improves in the first 2 years after surgery but deteriorates in the following 5–6 years [190]. A systematic review that included the STEP, SUSTAIN, PIONEER, and STAMPEDE (Surgical Treatment and Medications Potentially Eradicate Diabetes Efficiently) trials found that semaglutide led to significant weight loss, although it was associated with some adverse effects, such as gastrointestinal (GI) disturbances and infrequent occurrences of thyroid cancer. The long-term effects indicated that there was a partial regain of weight and a return of certain cardiometabolic parameters to baseline levels following the cessation of the medication [191]. In contrast, bariatric surgery, as demonstrated in the Longitudinal Assessment of Bariatric Surgery (LABS) consortium and supported by findings from the STAMPEDE trial, showed greater efficacy in promoting weight loss and managing obesity-related complications like diabetes. Specifically, bariatric procedures such as Roux-en-Y gastric bypass (RYGB) and sleeve gastrectomy (SG) resulted in a significantly higher proportion of patients reaching their diabetes treatment goals [192].

Although bariatric surgery demonstrates greater efficacy, it is associated with a higher risk of both early and long-term complications. In contrast, semaglutide offers a non-invasive alternative to surgery that achieves significant weight loss with a lower incidence of adverse effects.

We can conclude that consideration of the additional beneficial effects of GLP-1RA, such as cardiovascular and renal risk reduction, stroke risk reduction, beneficial effects on NASH, possible anti-inflammatory effects on perivascular adipose tissue, and a reduction in the incidence of prediabetes, anti-obesity medications may outweigh the use of metabolic surgery, especially given the known complications of invasive measures. On the other hand, bariatric surgery and anti-obesity medications can complement each other, particularly for individuals not achieving adequate weight loss with only one of these treatment options.

## What are the economic challenges and potential cost benefits of bariatric surgery?

Limited data exists on the economic impact of bariatric surgery in the MENA region. The decision on bariatric surgery necessitates a thorough evaluation of both financial and health-related considerations. The long-term advantages of the procedure sometimes surpass the upfront financial costs.

In Egypt, as an example of MENA region countries, it is crucial to promptly tackle the problem of poverty, especially following the 2011 revolution, which underscored the importance of social justice. A World Bank report from 2007 revealed that roughly 40% of the Egyptian population lives in poverty.

Recent results from the Egyptian Family Observatory Survey depict a complex situation. The survey shows that although 80% of households have at least one member enrolled in public health insurance, only 25% actually receive benefits from it. The quality of health services is often poor, and various administrative hurdles hinder access to care. This scenario is not just about numbers; it poses a serious risk to the financial stability of many families. As health issues increase, there is a growing danger that many average Egyptians will fall into poverty, worsening an already dire situation [193].

In 2008, healthcare funding mainly came from out-of-pocket costs, which represented 60% of total health expenditures. A significant share of these costs, totaling 39%, was directed towards private clinics, while 33% was spent on medications. Due to shortcomings in public healthcare, families often turn to expensive private healthcare options, as the national health insurance system fails to meet patients’ needs or shield them from health-related financial risks [194, 195]. On the other hand, there is a possible path to improvement: bariatric surgery. This treatment is available to both teenagers and adults who satisfy certain Body Mass Index (BMI) criteria. For those dealing with obesity, this procedure can greatly improve their quality of life. This demonstrates that effective healthcare solutions can be found within Egypt’s complex medical system. Now is a favorable time to push for reforms that will reduce poverty and enable families to make healthier choices [196].

Numerous factors affect the cost of bariatric surgery in Egypt. These factors include medical tests and scans, psychological assessments, the surgeon’s expertise, the patient’s health status and degree of obesity, the hospital’s reputation, geographical location, the type of surgery performed, postoperative care, hospital room quality, length of stay, medication costs after surgery, possible postoperative complications, and the length of rehabilitation and recovery. The surgery itself represents a substantial part of the overall cost, which includes the medical team’s expertise, operating room expenses, anesthesia, and medical supplies [197].

The price of bariatric surgery in Egypt is particularly competitive when compared to other nations. Many people opt for Egypt as a medical tourism destination due to its affordability and high medical standards, often reporting favorable results. In Egypt, the average cost of bariatric surgery falls between $3,000 and $5,000, whereas the costs are $4,000 in Turkey, $6,000 in India, $8,700 in the UAE, and $25,000 in the USA. Despite a global medical inflation rate of about 10.3%, costs in Egypt have not risen as expected. Data from a survey of three Egyptian surgeons may be underestimated, as both primary (expert interviews) and secondary (publications and payer service lists) sources were used to assess the cost per patient [198–200].

Several financing options are available in Egypt to improve access to bariatric surgery. These options consist of payment plans, medical loans, and health insurance coverage. Nonetheless, many insurance companies are hesitant to cover bariatric surgery costs. While some may agree to cover specific surgeries or related expenses, it is essential to verify coverage with the provider in advance, as many plans come with particular requirements. Moreover, some hospitals and clinics provide financing alternatives to help manage costs, which may include installment plans and medical loans. The majority of expenses related to bariatric surgery in Egypt are covered out of pocket, which can cause financial strain and potentially push families into poverty if these costs surpass 40% of the household’s payment capacity. As a result, low- and middle-income patients with severe obesity often find themselves excluded from bariatric surgery options in Egypt. Thus, cost remains a significant obstacle to the adoption of this medical technology. Creating strategies to reduce expenses is vital for enhancing the cost-benefit ratio of metabolic and bariatric surgery in Egypt [201, 202].

## Recommendations: AASD

1. The AASD recommends lifestyle changes in the form of a reduced-calorie diet and increased physical activity, together with new anti-obesity medications, including GLP-1 RA and dual GIP/GLP-1 receptor agonists, as first-line treatment for patients with obesity, prediabetes, and type 2 diabetes. Anti-obesity medications are to be used for weight management in patients with a BMI of ≥ 30 kg/m² or ≥ 27 kg/m² associated with one or more weight-related comorbidities.
2. AASD recommends improving public and decision-makers’ understanding of MBS indications through general media and enhancing healthcare providers’ knowledge with additional workshops.
3. AASD recommends thorough patient education, including detailed consent forms, and urges health authorities to mitigate risks to improve patient safety and outcomes.
4. AASD advises that patients undergo comprehensive preparation for MBS. The multidisciplinary team including a surgeon, internist or endocrinologist, registered dietitian, psychiatrist, psychologist, anesthetist, and gastroenterologist should collaboratively determine whether surgery is an appropriate treatment option.
5. AASD highlights the importance of psychiatrists and psychologists as part of the multidisciplinary team for MBS. They should provide psychological assessments and support before and after the procedure to evaluate readiness and ensure well-being.
6. AASD supports MBS or patients with a BMI over 40 kg/m² if preoperative criteria are met; for those with a BMI > 35 kg/m² with one or more severe obesity-related comorbidities; and for individuals with a BMI of 30–34.9 kg/m² with poorly controlled type 2 diabetes despite optimal medical management.
7. AASD suggests that MBS for diabetes remission be considered for patients with a diabetes history of less than 8 years, provided they meet BMI criteria.
8. AASD views MBS as a weight-loss option for younger individuals with prediabetes who meet BMI criteria, aiming to prevent diabetes progression.
9. AASD advises cautious use of MBS in selected adolescents with type 2 diabetes, fulfilling criteria for MBS, weighing the benefits against risks and complications. A multidisciplinary team should assess eligibility and oversee pre- and postoperative care.
10. AASD does not recommend MBS for patients with type 1 diabetes, except for those with severe obesity and good metabolic control, after a trial of anti-obesity medications for 6 months at least. Instead, the focus should remain on nutrition, lifestyle adjustments, and pharmacotherapy if needed, as potential risks may outweigh the benefits.
11. AASD does not recommend MBS specifically for NAFLD but suggests it as an option for patients who qualify for weight loss surgery. Large-scale, long-term randomized trials are required to provide clearer evidence.
12. AASD advises that while MBS may improve kidney and cardiovascular outcomes, it should be carefully considered due to potential risks. It is best suited for individuals with a BMI over 35 kg/m² who have not responded to intensive medical therapy.
13. AASD recommends that the choice of MBS procedure be made by a multidisciplinary team, taking all relevant factors into account.
14. AASD recommends that patients undergo lifestyle counseling, including guidance on physical activity, medical nutritional therapy (with a short-term liver-reducing diet), and smoking cessation for an adequate period before MBS.
15. AASD emphasizes the importance of recognizing and addressing potential long-term complications of MBS. Short- and long-term follow-ups are essential, including access to a dietitian and psychologist in addition to the surgeon. Nutritional follow-up should be maintained for at least two years, with nutritional blood tests and adherence to vitamin and mineral supplements. Long-term annual follow-up is mandatory.
16. AASD recommends individualized decisions for revisional MBS based on comprehensive assessments, including nutritional and psychological evaluations. GLP-1RA and dual GIP/GLP-1receptor agonists, should be considered for weight management before proceeding with revisional surgery.
17. AASD calls for long-term studies to assess the lasting benefits and cost-effectiveness of MBS for diabetes compared to lifestyle and medical treatments. While current evidence highlights short-term benefits, long-term trials are needed.
18. AASD advocates for the establishment of national or regional registries for MBS to assess its long-term effectiveness and safety.

## Data Availability

No datasets were generated or analysed during the current study.
